# Serum Uric Acid Predicts Declining of Circulating Proangiogenic Mononuclear Progenitor Cells in Chronic Heart Failure Patients

**DOI:** 10.15171/jcvtr.2014.004

**Published:** 2014-09-30

**Authors:** Alexander E. Berezin, Alexander A. Kremzer, Tatyana A. Samura, Tatyana A. Berezina, Yulia V. Martovitskaya

**Affiliations:** ^1^State Medical University, Internal Medicine Department, Zaporozhye, Ukraine; ^2^State Medical University, Clinical Pharmacology Department, Zaporozhye, Ukraine; ^3^Privat Medical center “Vitacenter”, Zaporozhye, Ukraine; ^4^State Medical University, Pathology Department, Zaporozhye, Ukraine

**Keywords:** Chronic Heart Failure, Serum Uric Acid, Circulating Mononuclear Progenitor Cells, Predictive Value

## Abstract

*Introduction:* Serum uric acid (SUA) is considered a marker for natural progression of chronic heart failure (CHF) mediated cardiovascular remodelling. CHF associates with declining of circulating mononuclear progenitor cells (MPCs). The objective of this study was to evaluate the interrelationship between SUA concentrations and proangiogenic MPCs in ischemic CHF patients.

*Methods: * The study population was structured retrospectively after determining the coronary artery disease (CAD) by contrast-enhanced spiral computed tomography angiography in 126 subjects with symptomatic ischemic mild-to-severe CHF and 128 CAD subjects without CHF. Baseline biomarkers were measured in all patients. Cox proportional multivariate hazard ratio was calculated for predictors of MPCs declining in both CHF and non-CHF patient population predictors of MPCs declining in CHF subjects were examined in stepwise logistic regression. C-statistics, integrated discrimination indices (IDI) and net-reclassification improvement were utilized for prediction performance analyses.

*Results:* Cox proportional adjusted hazard ratio analyses for CD14^+^CD309^+^ and CD14^+^CD309^+^Tie2^+^ MPCs by SUA has shown that the higher quartiles (Q3 and Q4) of SUA compared to the lower quartiles (Q1 and Q2) are associated with increased risks of depletion of both CD14^+^CD309^+^ and CD14^+^CD309^+^Tie2^+^ MPCs. The addition of Q4 SUA to the ABC model improved the relative IDI by 13.8% for depletion of CD14^+^CD309^+^ MPCs and by 14.5% for depletion of CD14^+^CD309^+^Tie2^+^ MPCs.

*Conclusion: * Circulating levels of proangiogenic MPCs are declined progressively depending on the levels of SUA in the HF subjects with CHF. We suggest that even mild elevations of SUA might be used to predict of relative depletion of proangiogenic MPCs among chronic HF patients.

## Introduction

Chronic heart failure (CHF) has remained as a potentially fatal complication of any cardiovascular diseases.^1^ Serum uric acid (SUA) has been shown to be an independent predictor of outcome in the general population and in patients with HF, the metabolic syndrome, type-2 diabetes mellitus (T2DM), chronic kidney disease and atherosclerosis.^2^ Although sustained hyperuricemia is considered an independent factor that is adversely associated with a higher mortality, however its causal role is yet to be established in CHF.^3^ Meta-analysis provided by Huang et al suggested that existing hyperuricemia was associated with 65% increases in the risk of suffering from HF (HR 1.65; 1.41–1.94) and that for every 1 mg/dL increase in SUA, the odds of developing CHF increased by 19% (HR 1.19; 1.17–1.21).^4^ Tamariz et al reported that SUA predicts all-cause mortality in CHF.^5^ There is a controversy in the causal role of hyperuricemia in HF.^6^

Less is known about the association between SUA levels and circulating mononuclear progenitor cells (MPCs). These cells possess angiopoetic function and are crucial in tissue repair.^7^ It has been shown that MPCs are recruited by the proinflammatory cytokines that are produced in patients with CHF.^8,9^ Substantially, many studies have demonstrated that circulating MPCs are declined progressively depending severity of CHF.^10-12^ We postulate that depletion of MPCs in the peripheral blood may link SUA to the inflammatory responses and the clinical outcome of patients with CHF. Therefore, CD34^+^ MPC count is not associated with cardiovascular remodelling or clinical outcome in CHF patients.^12,13^

Recent evidence suggests that the number of circulating proangiogenic CD14^+^CD309^+^ and CD14^+^CD309^+^Tie^+2^ MPCs is decreased in patients with compensated CHF, however, a linear relation between the number of these cells and SUA level has yet to be investigated.^14^ the objective of this study was to evaluate such a relationship between SUA and the number of proangiogenic population of MPCs in the peripheral blood of the patients with ischemic CHF.

## Materials and methods

### Study population

The study population was structured retrospectively after determining the coronary artery disease (CAD) by contrast-enhanced spiral computed tomography angiography in 126 subjects with symptomatic ischemic mild-to-severe CHF and 128 CAD subjects without HF. CHF was diagnosed according to the current clinical guidelines.^15^ All patients consented to voluntary participation in the study. Patients were excluded if they had a recent (within 3 months prior to study enrolment) myocardial infarction (MI) with or without Q-wave. Additional exclusion criteria included severe kidney disease with serum creatinine >440 μmol/L or estimated glomerular filtration rate (calculated using MDRD-6 formula^16^ ) <35 ml/min/m^2^ ;liver diseases that may affect clinical outcomes; malignancy; brain injury within 3 months prior to study enrolment; pulmonary edema; tachyarrhythmias; valvular heart disease; thyrotoxicosis; ischemic stroke; intracranial haemorrhage; acute infections; surgery; trauma; inflammation within a previous month; neoplasm; pregnancy; implanted pacemaker, and patient’s refusal to participate in the study.

### Investigation of coronary arteries

Multiplex spiral computed tomographic angiography was carried out to verify the ischemic origin of CHF for all patients prior to their inclusion in the study. When atherosclerotic lesions of the coronary arteries were verified, patients were subjected to conventional angiographic examination provided indications for revascularization were available. Diagnosis of CAD was made only by angiographic exams carried out within 6 months of the study. The coronary artery wall structure was measured while patients were holding their respiration. All measurements were obtained by means of contrast spiral computed tomographic angiography on Somatom Volum Zoom^®^ scanner (Siemens, Erlangen, Germany) with two detector rows.^17^ After preliminary native scanning, non-ionic contrast Omnipak (Amersham Health, Ireland) was administered for the optimal image of the coronary arteries. To reconstruct the image, 0.6-mm-width axial tomographic slices were used.

### Echocardiographic examination

A two-dimensional transthoracic echocardiography was performed for all patients using Acuson^®^ (Siemens, Germany) system with a 5 МHz transducer. Routine B-mode images and Doppler measurements were obtained both at the bedside and by post processing. Left ventricular end-diastolic and end-systolic volumes were measured by modified Simpson’s method. Left ventricular ejection fraction (LVEF) was assessed in compliance with the requirements of American Society of Echocardiography.^18^ Tissue Doppler echocardiography was carried out in 4-, 3- and 2-chamber projections in each of 16 segments of the left ventricle and in 4 spots of the mitral annulus: at the base of posterior septal, lateral, inferior, and anterior left ventricular walls.^18^ Peak systolic (Sm), early diastolic (Em), and late diastolic (Аm) myocardial velocities were measured in the mitral annulus area, followed by calculating velocity of early diastolic left ventricular filling (E) to Аm (Е/Аm) ratio and to Em (Е/Em) ratio.^19^

### Blood Sampling and Biochemical Measurements

Fasting venous blood samples were collected into silicone test tubes. They were centrifuged upon cooling at 6,000 rpm 430 g for 3 minutes. Then, plasma was refrigerated immediately and stored at a temperature no higher than -35 ^º^С until they were analysed. Samples were processed according to the recommendations of the manufacturer of the analytical technique. SUA level was measured by enzymatic methods using a chemical analyzer Beckman (Synchron LX20, city, state). Analytical range average for SUA was 0.5-82 mmol/L. N-terminal pro-brain natriuretic peptide (NT-pro-BNP) was measured by immune-electro-chemiluminescence method using sets by R&D Systems (city, state, USA) on Elecsys 1010 analyzer (Roche, Mannheim, Germany). Calibration of the assay was performed according to the manufacturer’s recommendations and values were normalized to a standard curve. Concentrations of total cholesterol (TC) and high-density lipoprotein (HDL) cholesterol were determined with Dimension Clinical Chemistry System^®^ (Dade Behring Inc, Newark, NJ). Low-density lipoprotein (LDL) cholesterol was calculated using Friedewald formula.^20^

### Flow Cytometry and cytologic experiments

High-Definition Fluorescence Activated Cell Sorter (HD-FACS) was used to detect circulating cells subsets depending on the expression of CD45, CD34, CD14, Tie-2, and VEGFR2, as previously described.^21^ Accordingly, the cells in question were labeled on the basis of their forward scatter characteristic (FSC) and side scatter characteristic (SSC) profiles. The cells were directly stained and analysed for the phenotypic expression of surface proteins using anti-human monoclonal antibodies, including anti-CD45 FITC (BD Biosciences, USA), anti-CD34 FITC (BD Biosciences, USA), anti-VEGFR-2 known as anti-CD309 FITC (BD Biosciences, USA), anti-Tie2 (BD Biosciences, USA) and anti-CD14 (BD Biosciences, USA) (For all cases labelling up to 2 × 10^9^ cells). The fluorescence minus one technique was used to provide negative controls and establish positive stain boundaries. After lysis of erythrocytes with Utilize^®^ wash solution, the samples were centrifuged at 200 g for 15 min; then they were washed twice with phosphate buffer solution and fixed immediately. Double- or triple-positive events were determined using Boolean principles (‘and’, ‘not’, ‘or’, etc.). Circulating endothelial progenitor cells (EPCs) are defined as CD34/VEGFR2 positive cells in lack of CD45 expression. 500,000 events were analyzed from each tube. For CD14^+^ populations, co-expression with Tie-2- and/or VEGFR-2- was determined using quadrant analysis. Standardized cell counts were presented as a percentage of total white blood cells count, which were identified as the total number of all CD45^+^ cells.

### Statistical Analysis

Statistical analysis of the results obtained was carried out in SPSS system for Windows, Version 20 (SPSS Inc, Chicago, IL, USA). The data were presented as mean and the standard errors of means or 95% confidence interval (CI); median and interquartile range (IQR). To compare categorical variables between groups, Chi squaretest (χ^2^ ) and Fisher’s exact test were used. The circulating NT-pro-BNP and SUA level in the blood failed to have a normal distribution, while distribution of the TG (triglycerides) and cholesterol fractions had a normal character (estimated by means of Kolmogorov-Smirnov test) and was not subjected to any mathematical transformation. Kruskal–Wallis test was used for difference in medians across quartiles of SUA. For further analysis concentrations of SUA in CHF patients were distributed by quartiles: QI (less 22.33 mmol/L), QII (23.2 to 31/10 mmol/L), QIII (32.0 to 39.0 mmol/L), QIV (40.00 to 49.60 mmol/L). QI=20.11 (19.0622.33) mmol/L; QII=27.53 (23.2; 31.10) mmol/L; QIII=35.80 (32.0; 39.0) mmol/L; and QIV=44.9 (40.00; 49.60) mmol/L. The factors, which could be associated potentially with MPCs declining, were determined by univariate and then multivariate regression analysis. Cox proportional multivariate odds ratio (OR) and 95% CI were calculated for predictors of MPCs declining in CHF and non-CHF patient population. Receiver operating characteristic (ROC) curves was configured to establish cut-off points of SUA level that optimally predicted decreased MPCs. Predictors of MPCs declining were examined in stepwise logistic regression with subjects with complications at the time of enrollment excluded from analyses. C-statistics, integrated discrimination indices (IDI) and net-reclassification improvement (NRI) were utilized for prediction performance analyses in CHF patients. A calculated probability of less than 0.05 was considered significant.

## Results

### General characteristics of study patient population

Table 1 shows a general characteristic of the patients included in the study. There were no substantial age and gender differences between the groups. The functional class of the patients with CHF were almost equally distributed from NYHA class I through NYHA class III (30.2%; 38.1%; and 31.7% respectively). Co-morbid conditions were dyslipidaemia (44.4%), hypertension (66.7%) and T2DM (36.5%). Additionally, CHF and non-CHF patients had similar cardiovascular risk, biochemical biomarkers and hemodynamic characteristics. As expected, circulating NT-pro-BNP was higher in CHF patients when compared with CHF-free CAD subjects. SUA levels for subjects with CHF (31.0; 22.8-41.9 mmol/L) were similar to those without CHF (28.5; 20.2-37.6 mmol/L, P=0.26).

**Table 1:** General characteristics of study patient population

	**Patients without CHF** **(n=128)**	**Patients with CHF (n=126)**					**P value**
		**Entire cohort patients with CHF**	**Quartile I (19.06-22.33 mmol/L)**	**Quartile II (23.2-31.10 mmol/L)**	**Quartile III (32.0-39.0 mmol/L)**	**Quartile IV (40.00 - 49.60 mmol/L**	
Age, years	57.80±6.38	58.34±9.60	57.70±6.10	57.40±6.76	60.30±4.20	62.60±6.22	0.42
Male, n (%)	72 (56.3%)	74 (58.7%)	17 (44.7%)	21 (65.6%)	18 (66.7%)	18 (62.0%)	0.28
Framingham General Cardiovascular Risk, %	22 (16-26)	23 (16 - 27)	22 (15 - 26)	24 (16-30)	23 (17-31)	23 (16-30)	0.75
NYHA class I, n (%)	-	38 (30.2%)	11 (29.0%)	9 (28.1%)	8 (29.6%)	10 (34.4%)	0.42
NYHA class II, n (%)	-	48 (38.1%)	14 (36.8%)	12 (37.5%)	10 (37.0%)	12 (41.4%)	0.44
NYHA class III, n (%)	-	40 (31.7%)	13 (34.2%)	11 (34.4%)	9 (33.3%)	7 (24.1%)	0.48
Hypertension, n (%)	81 (63.3%)	84 (66.7%)	25 (65.8%)	22 (68.8%)	18 (66.7%)	19 (65.5%)	0.86
Dyslipidaemia, n (%)	52 (40.6%)	56 (44.4%)	17 (44.7%)	15 (46.9%)	12 (44.4%)	12 (41.3%)	0.79
T2DM, n (%)	48 (37.5%)	46 (36.5%)	14 (36.8%)	12 (37.5%)	10 (37.0%)	10 (34.5%)	0.80
Premature CAD, n (%)	14 (10.9%)	12 (9.5%)	3 (7.9%)	3 (9.3%)	2 (7.40%)	4 (13.9%)	<0.01
Smoking, n (%)	28 (21.8%)	26 (20.6%)	8 (21.0%)	6 (18.8%)	5 (18.5%)	7 (24.1%)	0.42
BMI, kg/m^2^	23.6 (95% CI 21.8–27.6)	24.1 (95% CI 21.6 – 28.7)	23.3 (95% CI 20.1–25.1)	25.0 (95% CI 20.8–27.2)	24.6 (95% CI 19.4–25.9)	25.2 (95% CI 19.5–25.5)	0.58
Mean systolic BP, mm Hg	131.20±4.66	130.90±8.41	127.30±5.98	133.80±6.12	129.20±6.34	128.10±4.93	0.44
Mean diastolic BP, mm Hg	76.10±3.97	77.24±4.22	76.85±3.15	77.17±3.21	77.10±3.11	77.70±2.95	0.42
Heart rate, beat per min	69.42±3.88	70.52±3.34	68.56±5.11	70.44±5.68	71.36±4.66	70.16±5.12	0.52
LV EF, %	50.40±2.25	43.80±0.77	44.10±0.94	43.50±0.97	43.60±0.79	43.10±0.85	0.28
Е/Аm, U	15.1±1.13	16.6±0.94	16.3±0.82	16.5±0.76	16.5±0.82	17.1±0.72	0.48
Е/Em, U	15.4±1.22	16.6±1.00	16.2±0.89	16.6±0.72	17.2±0.55	17.0±0.56	0.46
eGFR, mL/min/m^2^	88.5 (95% CI=66.1–112.3)	82.3 (95% CI=68.7–102.6)	93.5 (95% CI=88.3–100.3)	86.1 (95% CI=68.3–104.1)	83.5 (95% CI=68.3–112.6)	76.2 (95% CI=61.1–98.3)	0.045
HbA1c, %	6.5 (95% CI=4.3–8.7)	6.8 (95% CI=4.1-9.5)	6.8 (95% CI=3.9–8.9)	6.9 (95% CI=3.5–9.6)	6.8 (95% CI=3.7–8.9)	6.9 (95% CI=3.8–9.2)	0.86
Fasting glucose, mmol/L	5.10 (95% CI=3.5–8.3)	5.20 (95% CI=3.3-9.1)	5.11 (95% CI=3.2–8.5)	5.28 (95% CI=3.1–8.9)	5.21 (95% CI=3.0–9.5)	5.17 (95% CI=3.2–9.0)	0.87
Creatinin, μmol/L	70.6 (95% CI=53.5–92.5)	72.3 (95% CI=58.7–92.6)	70.7 (95% CI=53.1–98.5)	71.1 (95% CI=55.7–108.2)	73.7 (95% CI=53.8–109.5)	88.1 (95% CI=63.0–134.2)	0.048
TC, mmol/L	5.3 (95% CI=4.1–6.2)	5.1 (95% CI=3.9-6.1)	5.0 (95% CI=3.7–6.4)	5.1 (95% CI=3.8–6.3)	5.0 (95% CI=3.9–6.0)	5.0 (95% CI=3.7–6.2)	0.12
HDL cholesterol, mmol/L	0.95 (95% CI=0.90–1.07)	0.91 (95% CI=0.89-1.12)	0.95 (95% CI=0.92–1.14)	0.94 (95% CI=0.88–1.12)	0.91 (95% CI=0.86–1.13)	0.90 (95% CI=0.83–1.10)	0.12
LDL cholesterol, mmol/L	3.34 (95% CI=3.20–4.64)	3.23 (95% CI=3.11-4.4)	2.95 (95% CI=2.84–4.6)	3.15 (95% CI=2.90–4.6)	3.24 (95% CI=3.01–4.7)	3.265 (95% CI=2.98–4.64)	0.64
NT-pro-BNP, pg/mL	243.7 (95% CI=149.8–369.5)	1533.6 (95% CI=644.5–2560.6)	1263.9 (95% CI=688.2–2120.4)	1446.2 (95% CI=612.6–2873.5)	1590.6 (95% CI=622.4–2710.2)	1873.5 (95% CI=711.2–2790.4)	0.22

Note: CI, confidence interval; T2DM, type 2 diabetes mellitus; eGFR, estimated glomerular filtration ratio; BMI, body mass index; TC, total cholesterol; HbA1c, glycated haemoglobin; LDL, low-density cholesterol; HDL, high-density cholesterol; BP, blood pressure; LVEF, left ventricular ejection fraction; U, unit; Em, early diastolic myocardial velocity; Аm, late diastolic myocardial velocity; E, peak velocity of early diastolic left ventricular filling.

There was no difference in gender, age and body habitus distribution among SUA quartiles of patients with CHF. However, in patients with no evidence of CHF, SUA concentrations were significantly higher in men (Me= 29.9; 24.5-37.8 mmol/L) than those in women (Me= 23.1; 19.8-30.1 mmol/L, P=0.04). The prevalence of comorbidities; cardiovascular risk, hemodynamic parameters NT-pro-BNP and the NYHA functional class; serum creatinine concentrations and eGFR values; fasting blood glucose concentrations and HbA1c percentages were similar among different quartiles of SUA. The prevalence of premature CAD was higher in 4^th^ quartile of SUA when compared to other quartiles (P<0.05). No significant difference was found in anatomic distribution of the stenotic coronary arteries and related atheromatous lesions among the SUA quartiles.

Baseline angiographic and treatment characteristics of study patient population are presented in Table 2. Among patients with CHF; 36.5% had single vessel; 33.3% had double vessel and the remaining 20.2% had triple vessel disease. Treatment strategies were according to the current clinical guidelines and were similar in all subgroups ACEI/ARBs and aspirin were given for all patients across SUA quartiles in similar proportions. Compared with the first quartile SUA cohort, patients with QII-IV SUA cohorts had a higher prescribing rate of beta-blockers, anti-aldosterone diuretics (P<0.05), but lower prescribing rate of channel blockers and statins (P<0.05).

**Table 2:** Baseline angiographic and treatment characteristics of study patient population

**Variables**	**Patients without CHF (n=128)**	**Entire cohort patients with CHF (n=126)**	**Quartile I (19.06-22.33 mmol/L)**	**Quartile II (23.2 - 31.10 mmol/L)**	**Quartile III (32.0 - 39.0 mmol/L)**	**Quartile IV (40.00 - 49.60 mmol/L)**	**P**
Coronary arteries with plaques determined							
1 vessel, n (%)	48 (37.5%)	46 (36.5%)	12 (31.6%)	13 (40.6%)	11 (40.7%)	10 (34.5%)	0.66
2 vessels, n (%)	46 (35.9%)	42 (33.3%)	13 (34.2%)	10 (31.3%)	9 (33.3%)	10 (34.5%)	0.72
3 vessels and more, n (%)	34 (26.6%)	38 (30.2%)	13 (34.2%)	9 (28.1%)	7 (25.9%)	9 (31.0%)	0.73
Medications							
ACEI/ARBs, n (%)	116 (90.6%)	126 (100%)	38 (100%)	32 (100%)	27 (100%)	29 (100%)	0.52
Aspirin, n (%)	101 (78.9%)	98 (77.8%)	31 (81.6%)	25 (65.8%)	22 (81.5%)	20 (69.0%)	0.54
Other antiagregants, n (%)	27 (21.1%)	6 (4.8%)	2 (5.2%)	1 (3.1%)	1 (3.7%)	2 (6.9%)	0.86
Beta-blockers, n (%)	111 (86.7%)	104 (82.5%)	16 (42.1%)	32 (100%)	27 (100%)	29 (100%)	<0.05
Ivabradin, n (%)	36 (28.1%)	37 (29.4%)	22 (57.9%)	12 (37.5%)	2 (7.4%)	1 (3.4%)	<0.05
Mineralocorticoid antagonists, n (%)	-	52 (41.3%)	4 (10.5%)	19 (59.4%)	14 (51.9%)	15 (51.7%)	<0.05
Diuretics, n (%)	81 (63.3%)	106 (84.1%)	19 (50.0%)	25 (78.1%)	27 (100%)	29 (100%)	<0.05
Statins, n (%)	128 (100%)	94 (74.6%)	33 (86.8%)	28 (87.5%)	22 (81.5%)	11 (37.9%)	<0.05
Metformin, n (%)	43 (33.6%)	41 (32.5%)	9 (23.7%)	11 (34.3%)	12 (44.4%)	9 (31.0%)	0.054

Note: CI, confidence interval; ACEI, angiotensin-converting enzyme inhibitor; ARBs, angiotensin-2 receptor blockers.

### Circulating MPCs level in the study patient population

Table 3 shows the prevalence of various phenotypes of CD34^+^MPCs in the peripheral blood. Subjects with higher SUA quartile had significantly lower MPCs counts when compared with patient with low quartiles. CD45^+^CD34^+^ cell count was positively correlated to the LVEF (r=0.686; P=0.001), negatively correlated to the Е/Аm ratio (r=-0.566; P=0.001), the Е/Еm ratio (r=-0.568; P=0.001). CD45^+^CD34^+^ cell count was also correlated negatively to eGFR (r=-0.561; P=0.025), SUA (r=-0.482; P<0.001), and the NT-pro-BNP concentrations (r=-0.353; P<0.001).

**Table 3:** Concentrations of MPCs in study patient population

**Cell phenotypes**	**Patients without** ** CHF (n=128)**	**Patients with CHF (n=126)**					**P**
		**Entire cohort patients** **with CHF (n=126)**	**Quartile I (19.06-** **22.33 mmol/L)**	**Quartile II (23.2-** **31.10 mmol/L)**	**Quartile III (32.0** **-39.0 mmol/L)**	**Quartile IV (40.00** ** - 49.60 mmol/L)**	
CD45^+^CD34^+^×10^-4^ , %	2.191 (1.76–2.613)	1.282 (1.21–1.528)	1.77 (1.58–1.93)	1.72 (1.53–1.91)	1.45 (1.21–1.68)	1.05 (0.80–1.17)	<0.01
CD45-CD34^+^×10^-4^, %	1.09 (1.00–1.348)	0.727 (0.54–0.913)	1.01 (0.91–1.15)	0.91 (0.81–1.01)	0.83 (0.72–0.93)	0.63 (0.33–0.86)	<0.01
CD14^+^CD309^+^ ×10^-4^, %	57.00 (43.20–81.50)	29.18 (15.00–34.50)	43.9 (33.7–54.12)	37.2 (28.8–45.59)	28.0 (17.48–37.2)	14.0 (11.1–19.86)	0.01
CD14^+^CD309^+^Tie^+2^×10^-4^, %	5.50 (3.05–8.15)	0.67 (0.21–1.10)	0.86 (0.74–0.98)	0.82 (0.73–0.92)	0.67 (0.58–0.76)	0.37 (0.29–0.56)	0.01

Note: The values correspond to medians and a interquartile range (IQR) of 25%–75%. Statistical comparisons are made using Mann-Whitney test with significance levels of 0.05 and 0.01 (for 2-tailed).

On the other hand, CD45-CD34^+^ cell count in the peripheral blood was negatively associated with T2DM (r= -0.614; P<0.001), SUA (r=-0.466; P<0.001), hypertension (r=-0.240; P=0.026), the NT-pro-BNP level (r=-0.605; P=0.002), eGFR (r=-0.423; P=0.012), active smoking (r=-0.222; P=0.040). A positive association was found between the CD45-CD34^+^ cell count and LVEF (r=0.723; P<0.001), the Е/Аm ratio (r=0.52; P=0.0024) and the Е/Еm ratio (r=0.60; P<0.001). The CD14^+^CD309^+^ subpopulation count was associated positively with LVEF (r=0.785; P<0.001), the Е/Еm ratio (r=0.52; P<0.001), the Е/Аm ratio (r=0.48; P<0.001); and it was associated negatively with the NYHA class (r=-0.622; P=0.001), T2DM (r= -0.521; P=0.001), SUA (r=-0.508; P=0.001), NT-pro-BNP level (r=-0.362; P=0.001), hypertension (r=-0.320; P=0.005), the total cholesterol level (r=-0.260; P=0.04), adherence to smoking (r=-0.259; P=0.042) and patient’s age (r=-0.254; P=0.002). The CD14^+^CD309^+^Tie2^+^ subpopulation count showed a positive association with LVEF (r=0.639; P=0.001), the Е/Еm ratio (r=0.52; P=0.001), eGFR (r=0.486; P=0.002); and a negative association with the NYHA class (r=-0.657; P=0.001), SUA (r=-0.628; P=0.001), T2DM (r=-0.610; P=0.001), the NT-pro-BNP level (r=-0.373; P=0.001), the serum concentrations of low**-**density lipoproteins (r=-0.354; P=0.001), the TG level (r=-0.258; P=0.043), adherence to smoking (r=-0.285; P=0.042), the body mass index (r=-0.272; P=0.046).

### Association between SUA level and biomarkers

SUA levels positively correlated with NYHA functional class (r=0.612; P<0.001); T2DM (r=0.462; P<0.001), NT-pro-BNP (r=0.612; P<0.001), diuretics (r = 0.37, P<0.01), body mass index (r= 0.34, P<0.05), hyperlipidemia (r= 0.32, P<0.05), age (r= 0.30, P<0.01), male gender (r= 0.29, P<0.05), and inversely correlated with eGFR (r= -0.476; P=0.002), LVEF (r= -0.42; P=0.001), CD45^+^CD34^+^ MPCs (r= -0.388; P=0.001); CD45-CD34^+^ MPCs (r= -0.41; P=0.001); CD14^+^CD309^+^ MPCs (r= -0.397; P=0.001); CD14^+^CD309^+^Tie2^+^ MPCs (r= -0.442; P=0.001). Multivariable linear regression analyses were performed for CD34^+^phenotypes of MPCs, adjusted for eGFR, BMI, LVEF, NYHA, diuretics, and T2DM. SUA level independently impacted the count of CD14^+^CD309^+^ cells (r= -0.388; P=0.001) and CD14^+^CD309^+^Tie2^+^ cells (r= -0.414; P=0.001), while it had no impact on the number of CD45^+^CD34^+^ cells (r= -0.214; P=0.22) and CD45-CD34^+^ cells (r= -0.16; P=0.16). The higher quartiles (Q3 and Q4) compared to the lower quartiles (Q1 and Q2) were associated with increased risk of cell depletion of both CD14^+^CD309^+^ and CD14^+^CD309^+^Tie2^+^ MPCs among CHF patients, while such a drop was not observed in non-CHF subjects (Table 4).

**Table 4:** Cox proportional adjusted Odds Ratios analyses for CD14^+^CD309^+^ and CD14^+^CD309^+^Tie2^+^ MPCs by SUA Quartiles

	**SUA Quartiles**	**SUA, mmol/L**		**Odds Ratio**	**95% CI**	**P value**
		**Median**	**95% CI**			
**For CHF patients**						
For CD14^+^CD309^+^ MPCs						
	Q1	20.11	19.06-22.33	1.00	-	-
	Q2	27.53	23.2-31.10	1.02	0.88-1.11	0.24
	Q3	35.80	32.0-39.0	1.18	1.06-1.29	0.001
	Q4	44.90	40.00-49.60	1.24	1.12-1.46	0.002
For CD14^+^CD309^+^Tie2^+^MPCs						
	Q1	20.11	19.06-22.33	1.00	-	-
	Q2	27.53	23.2-31.10	1.08	1.00-1.20	0.054
	Q3	35.80	32.0-39.0	1.22	1.11-1.34	0.001
	Q4	44.90	40.00-49.60	1.38	1.20-1.55	0.001
**For non-CHF patients**						
For CD14^+^CD309^+^ MPCs						
	Q1	20.21	18.18-22.50	1.00	-	-
	Q2	25.82	23.1-27.60	1.01	0.83-1.16	0.16
	Q3	30.60	28.3-32.7	1.04	0.92-1.15	0.12
	Q4	34.50	33.20-37.50	1.05	0.96-1.18	0.22
For CD14^+^CD309^+^Tie2^+^MPCs						
	Q1	20.21	18.18-22.50	1.00	-	-
	Q2	25.82	23.1-27.60	1.03	0.92-1.10	0.44
	Q3	30.60	28.3-32.7	1.06	0.95-1.12	0.32
	Q4	34.50	33.20-37.50	1.07	0.94-1.11	0.26

Note: All the respective biomarker-models are adjusted for eGFR, BMI, LVEF, NYHA, diuretics, and T2DM. Abbreviations: Q, quartile; MPCs, mononuclear progenitor cells; SUA, serum uric acid; eGFR, estimated glomerular filtration ratio; BMI, body mass index; LVEF, left ventricular ejection fraction; NYHA, New York Heart Association; T2DM, type two diabetes mellitus.

### The predictive value of SUA

**Figure 1:**
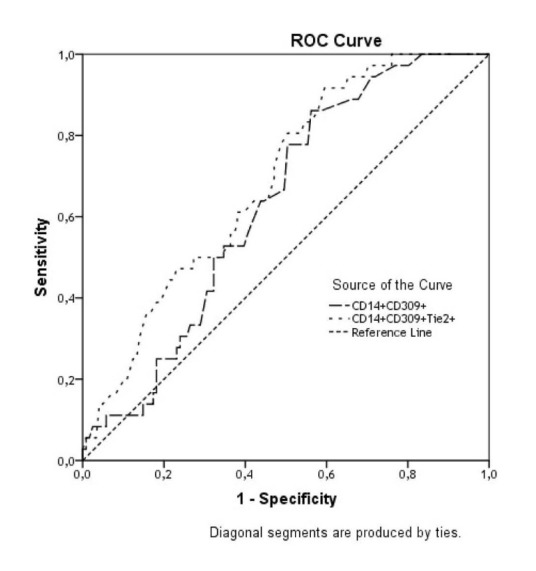
The predictive power of SUA in the both models for declining of CD14^+^CD309^+^ and CD14^+^CD309^+^Tie2^+^ MPCs in CHF patients. Results of the Receive Operation Characteristics analysis.

The predictive value of SUA level with respect to the MPCs with phenotypes CD14^+^CD309^+^ and CD14^+^CD309^+^Tie2^+^in the patients with CHF was performed using ROC-analysis, the results of which are presented in Figure 1. SUA demonstrated a high predictive value for declining both MPC phenotypes (CD14^+^CD309^+^ and CD14^+^CD309^+^Tie2^+^)in CHF patients. The estimated areas under the curves (AUC) were 0.631 (sensitivity= 63.9%; specificity= 56.2%) for CD14^+^CD309^+^ cells and 0.687 (sensitivity= 72.2%; specificity= 52.9%) for CD14^+^CD309^+^Tie2^+^ cell population. The cut-off value for the SUA level that predicted the cell loss best in both models was 31.5 mmol/L. Of all the variables (SUA, eGFR, BMI, LVEF, NYHA, diuretics, and T2DM) included in our multivariate model, SUA was the strongest predictor of proangiogenic MPCs depletion. However, the addition of SUA over cut-off point to the ABC model improved the relative IDI by 13.8% for depletion of CD14^+^CD309^+^ MPCs and by 14.5% for depletion of CD14^+^CD309^+^Tie2^+^MPCs (Table 5). For category-free NRI, 16% of events (P=0.001) and 19% of non-events (P=0.0001) were correctly reclassified by the addition of SUA to the ABC model for depletion of CD14^+^CD309^+^ cells (Table 6). When we added Q4 SUA to the ABC model, 18% of events (P=0.001) and 24% of non-events (P=0.0012) were reclassified for depletion of CD14^+^CD309^+^Tie2^+^cells. Thus, these data suggest that elevation of SUA might be considered a predictor of proangiogenic MPCs loss in patients with CHF.

**Table 5:** C-statistics for Models with SUA, eGFR, BMI, LVEF, NYHA, diuretics, and T2DM as Continuous Variables

**Models**	**AUC (95% CI)**	**ΔAUC**	**IDI (±SE)**	**Relative IDI (%)**
Depletion of CD14^+^CD309^+^ MPCs				
Model 1 (eGFR, BMI, LVEF, NYHA, diuretics, and T2DM)	0.528	-	-	-
Model 1 + Q4 SUA	0.664	-	-	-
Model 1 + Q4 SUA vs Model 1	-	0.136; P=0.001	0.05±0.015	13.8%
Depletion of CD14^+^CD309^+^Tie2^+^MPCs				
Model 1 (eGFR, BMI, LVEF, NYHA, diuretics, and T2DM)	0.516	-	-	-
Model 1 + Q4 SUA	0.672	-	-	-
Model 1 + Q4 SUA vs Model 1	-	0.156; P=0.001	0.06±0.012	14.5%

Note: Relative IDI – calculated as the ratio of IDI over the discrimination slope of the model without Q4 SUA (less than cut-off point equal 31.5 mmol/L of SUA). Abbreviations: AUC, area under curve; SE, standard error; IDI, integrated discrimination index; NRI, net reclassification improvement; MPCs, mononuclear progenitor cells; SUA, serum uric acid; eGFR, estimated glomerular filtration ratio; BMI, body mass index; LVEF, left ventricular ejection fraction; NYHA, New York Heart Association; T2DM, type two diabetes mellitus.

**Table 6:** Prediction performance analyses for models with SUA, eGFR, BMI, LVEF, NYHA, diuretics, and T2DM as continuous variables

**Model 2 vs model 1**	
Depletion of CD14^+^CD309^+^ MPCs	
Categorical NRI	0.11 (95% CI 0.07-0.14)
Percentage of events correctly reclassified	5 (P=0.11)
Percentage of non-events correctly reclassified	5 (P=0.012)
Categorical free NRI	0.42 (95% CI 0.26-0.54)
Percentage of events correctly reclassified	16% (P=0.001)
Percentage of non-events correctly reclassified	19% (P=0.0001)
Depletion of CD14^+^CD309^+^Tie2^+^MPCs	
Categorical NRI	0.14 (95% CI 0.10-0.19)
Percentage of events correctly reclassified	6 (P =0.14)
Percentage of non-events correctly reclassified	8 (P =0.010)
Categorical free NRI	0.48 (95% CI 0.29-0.62)
Percentage of events correctly reclassified	18% (P=0.001)
Percentage of non-events correctly reclassified	24% (P =0.0012)

Note: Model 1- eGFR, BMI, LVEF, NYHA, diuretics, and T2DM; Model 2 - eGFR, BMI, LVEF, NYHA, diuretics, T2DM and Q4 SUA. Abbreviations: NRI, net reclassification improvement; MPCs, mononuclear progenitor cells; SUA, serum uric acid; eGFR, estimated glomerular filtration ratio; BMI, body mass index; LVEF, left ventricular ejection fraction; NYHA, New York Heart Association; T2DM, type two diabetes mellitus.

## Discussion

Previous reports have been predominantly elucidated a relationship between cardiovascular outcomes and documented hyperuricemia in patients with acute and CHF.^3,22,23^ The effects of SUA on all-cause mortality at different SUA cut-off levels in CHF patient population were evaluated using meta-regression. There was a linear association between SUA after 7 mg/dL (41.6 mmol/L) and mortality.^24^ Arguing against a pure protective role of SUA in cardiovascular disease^25^ , we found that levels of SUA remained independently associated with depletion of proangiogenic MPCs after adjusting for parameters with known effects. These cells play critical role in tissue repair and neovascularization due to their angiogenic property. Elevated levels of SUA in CHF patient population were associated with progressively declining proangiogenic MPC.

These findings add to the existing controversy on the role of SUA in the evolution of CHF and its related outcomes. Elevated levels of SUA have been linked to the body habitus, diuretic use, declining eGFR, as wells as both biochemical (NT-pro-BNP) and hemodynamic (E/Ea and LVEF) markers of CHF by several investigators.^24,26^ Amin et al reported that even mild elevation of SUA is associated with impaired clinical and hemodynamic profile in patients with systolic CHF and suggested that SUA might be used as a non-invasive indicator of elevated left ventricular filling pressures.^27^ Misra et al demonstrated a reverse impact of CHF status (compensation or decompensation) on SUA levels among men with high cardiovascular risk profile.^28^ These investigators found that mild elevated SUA associated with increased risk of CHF decompensation (OR = 1.67; 95% CI 1.21 to 2.32).

Although hyperuricemia predominantly affects men, our findings failed to show differences in SUA between men and women diagnosed with CHF. According to our findings, there was no documented case of hyperuricemia (defined as SUA ≥6 mg/dL for women [35.6 mmol/L] and ≥8 mg/dL for men [47.6 mmol/L]). Moreover, none of our patients was treated for symptomatic hyperuricemia. However, the interpretation of SUA levels for individuals with CHF may be confusing, as even a small increase in SUA levels is probably viewed as a marker of endothelial dysfunction and an indicator of tissue repair in symptomatic CHF patients. Current evidence suggests that SUA could be a marker of oxidative damage in several settings associated with CHF, such as overweight, obesity, diuretic use. Although we described a modest linear association between SUA and body habitus and between SUA and diuretic use as we failed to show any correlation between these variables and the number of circulating MPCs.

It has predisposed that SUA may realize their antioxidative capacity. Therefore, it is not clear whether this increase in SUA levels may be an epiphenomenon, a counter-regulatory process or a detrimental factor that is contributing to the underlying pathophysiology. Since uric acid is a byproduct of purine catabolism and its serum levels increase as the enzymatic activity of xanthine oxidase surges in tissue hypoxia and subsequent apoptosis in CHF settings.^29^

The association between hyperuricemia and poor outcomes of CHF is more noticeable in patients without renal dysfunction than those with CKD.^29-31^ This finding suggests that hyperuricemia may predict poor outcomes when it primarily increased due to enhanced oxidative activity rather than the conditions where its renal excretion is impaired.^30^ Conversely Filippatos et al have found no association between SUA and BMI, as it is the case with our report.^29^ Diuretics, mainly the hydrochlorothiazides, are widely used to treat CHF, meanwhile elevating serum concentrations of uric acid by increasing the reabsorption of sodium and urate in the proximal tubule. Although we reported an association between SUA and diuretics administration, the direct effect of diuretics in depletion of MPCs in patient study population was not determined.

SUA may associate with CAD through its effect on coronary risk factors, such as obesity, hypertension, hypertriglyceridemia, dyslipidaemia and T2DM. Multivariate linear regression model that was constructed for CD34^+^phenotypes of MPCs demonstrated that even following an adjustment for eGFR, LVEF, NYHA functional class, diuretics, and T2DM, SUA had an independent impact of on the cell count of CD14^+^CD309^+^ MPCs and CD14^+^CD309^+^Tie2^+^ MPCs. We speculate that tissue ischemia results in enhanced oxidative activity of xanthine oxidase and subsequent production of uric acid. The observed suppression in recruitment, mobbing, differentiation and functional status of MPCs are mediated through Akt/STAT/MAP-kinase mechanisms as a reflection of chronic oxidative stress and probably, enhanced catabolic state in CHF.^32-34^ It is possible to address to new investigations whether relationships between SUA and proangiogenic MPCs are multidimensional, or if they can be associated with clinical outcomes.

## Conclusion

Circulating level of proangiogenic MPCs declines progressively depended on quartiles of SUA level in CHF subjects. We suggest that mild elevation of SUA (>31.5 mmol/L) might be considered a predictor of decline in the number of proangiogenic MPCs in patients diagnosed with CHF.

## Study restrictions

This study has some restrictions. The authors believe that a greater cohort is to be desirable to improve the power of the study. There is a variation in the definition of EPCs, the number of existing cardiovascular risk factors in various patients, and in the interaction between EPCs and other hematopoietic progenitor, inflammatory cells or platelets. The authors suppose that these restrictions might have no significant impact on the study data interpretation.

## Acknowledgements

We thank all patients for their participation in the investigation, staff of the Regional Zaporozhye Hospital (Ukraine) and the doctors, nurses, and administrative staff in City hospital # 6 (Zaporozhye, Ukraine), general practices, and site-managed organizations that assisted with the study.

## Ethical Issues

The study was approved by an institutional review committee. The investigators followed strictly all the requirements to clinical trials in conformity with the World Medical Association Declaration of Helsinki, 1964, 2008, good clinical practice provided by International Conference on Harmonization (GCP-ICH), Council of Europe Convention for the Protection of Human Rights and Dignity of the Human Being in view of using achievements in biology and medicine, convention on Human Rights and Biomedicine, including Additional Protocol to the Convention on Human Rights and Biomedicine, concerning Biomedical Research, and legislation of Ukraine.

## Competing interests

This research received no specific grant from any funding agency in the public, commercial, or not-for-profit sectors. The Authors declare that they have no competing interests.
